# Depauperate genetic variability detected in the American and European bison using genomic techniques

**DOI:** 10.1186/1745-6150-4-48

**Published:** 2009-12-08

**Authors:** Cino Pertoldi, Małgorzata Tokarska, Jan M Wójcik, Ditte Demontis, Volker Loeschcke, Vivi R Gregersen, David Coltman, Gregory A Wilson, Ettore Randi, Michael M Hansen, Christian Bendixen

**Affiliations:** 1 Mammal Research Institute, Polish Academy of Sciences, Waszkiewicza 1c, 17-230 Białowieża, Poland; 2 Department of Biological Sciences, Ecology and Genetics, Aarhus University, Ny Munkegade, Building 1540, 8000 Århus C, Denmark; 3 Department of Genetics and Biotechnology, Faculty of Agricultural Sciences, Aarhus University, PO Box 50, DK-8830 Tjele, Denmark; 4 Department of Biological Science, University of Alberta, Ctr Biol Sci, Edmonton, AB T6G 2E9, Canada; 5 Canadian Wildlife Service, 200, 4999 - 98 Ave, Edmonton, AB T6B 2X3, Canada; 6 Istituto Superiore per la Protezione e la Ricerca Ambientale, via Cá Fornacetta 9, I-40064, Ozzano Emilia (BO), Italy

## Abstract

A total of 929 polymorphic SNPs in EB (out of 54, 000 SNPs screened using a BovineSNP50 Illumina Genotyping BeadChip), and 1, 524 and 1, 403 polymorphic SNPs in WB and PB, respectively, were analysed. EB, WB and PB have all undergone recent drastic reductions in population size. Accordingly, they exhibited extremely depauperate genomes, deviations from genetic equilibrium and a genome organization consisting of a mosaic of haplotype blocks: regions with low haplotype diversity and high levels of linkage disequilibrium. No evidence for positive or stabilizing selection was found in EB, WB and PB, likely reflecting drift overwhelming selection. We suggest that utilization of genome-wide screening technologies, followed by utilization of less expensive techniques (e.g. VeraCode and Fluidigm EP1), holds large potential for genetic monitoring of populations. Additionally, these techniques will allow radical improvements of breeding practices in captive or managed populations, otherwise hampered by the limited availability of polymorphic markers. This result in improved possibilities for 1) estimating genetic relationships among individuals and 2) designing breeding strategies which attempt to preserve or reduce polymorphism in ecologically relevant genes and/or entire blocks.

**Reviewers:**

**This article was reviewed by: **Fyodor Kondrashov and Shamil Sunyaev

## Findings

Fifty-four EB (*Bison bonasus bonasus*), 30 WB (*Bison bison athabascae*) and 26 PB (*Bison bison bison*) were used for genome screening. The PB samples derive from Elk Island National Park while WB samples come from Wood Buffalo National Park (Canada). The relationships (mother, father and offspring) of three EB families were used in the SNP screening to verify Mendelian segregation. Genome-wide screening encompassed 54, 000 single nucleotide polymorphisms (SNPs) across the entire bovine genome. More than half of the SNPs were discovered using the sequencing system Genome Analyzer (Illumina^®^) [1]. The additional SNP content was derived from publicly available sources such as Btau (ftp://ftp.hgsc.bcm.tmc.edu/pub/data/Btaurus/fasta), the bovine reference genome, and the Bovine HapMap Consortium data set (). The BeadChip has an MAF of 0.25 across all loci and has been validated in both dairy and beef cattle. SNPs were genotyped on the BovineSNP50 BeadChip according to the Infinium II Multi-Sample assay protocol provided by Illumina^® ^(Manual Experienced User Card, 11208000 Rev. A., Illumina Inc.).

Despite the fact that the *Bos *and *Bison *lineages split about 1 million years ago [2], a total of 2, 209 polymorphic SNPs were found when EB, PB and WB were pooled. The average call rate for the bison samples was relatively high (96.60%) compared to the call rate found for the cattle samples (99.5%) (Illumina, Inc. Pub No. 370-2007-029) [1]. The small difference in call rate (2.9%) confirms the reliability of our comparisons and also reflects differences in the genomic DNA between the two groups such as the possibility of different alleles or deletions in SNPs.

The P% for each bison species was calculated relative to the total number of loci that were polymorphic when EB, WB and PB samples were pooled. The pairwise F_ST _used as a measure of differentiation between EB, PB and WB was estimated using FSTAT version 2.9.3 [3] (). Genetic differentiation between PB and WB was significant (F_ST _= 0.114), albeit much lower compared to EB-PB (F_ST _= 0.466) or EB-WB (F_ST _= 0.474). Genetic differentiation estimated between EB, WB and PB might provide a relative estimate of the time elapsed since divergence in isolation.

**Figure 1:**
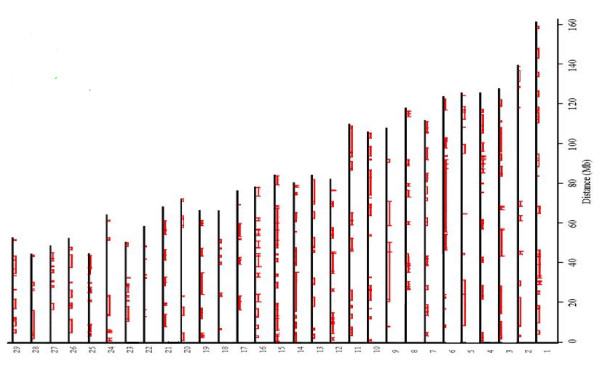
**The distribution of the 929 polymorphic SNPs in EB and haplotype block partitioning**. Hatch marks of all SNPs in a certain block are connected by a line. Each hatch represents a SNP.

**Figure 2:**
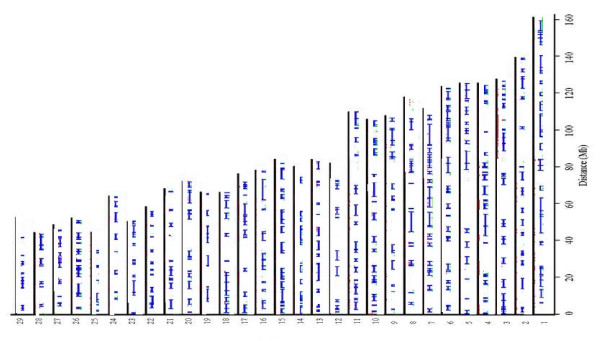
**The distribution of the 1, 524 polymorphic SNPs in WB and haplotype block partitioning**. Hatch marks of all SNPs in a certain block are connected by a line. Each hatch represents a SNP.

**Figure 3:**
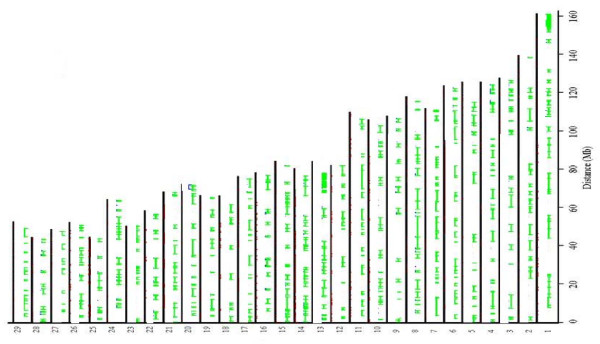
**The distribution of the 1, 403 polymorphic SNPs in PB and haplotype block partitioning**. Hatch marks of all SNPs in a certain block are connected by a line. Each hatch represents a SNP.

The number of mapped, polymorphic SNPs in EB was 929 (P% = 42%). The distribution of these markers along the autosomal chromosomes (on the corresponding known position on the 29 autosomal bovine chromosomes) is shown in Figure 1. More polymorphic SNPs were found in WB (P% = 69%) and PB (P% = 63%) (1, 524 and 1, 403 SNPs, respectively, see Figures 2 and 3).

After considering the distribution of the distance between the polymorphic loci the haplotypes were calculated according to the four gamete rule implemented in the Haploview 4.1 software [4].

Inspection of the polymorphic SNPs (see Figure 1, 2 and 3) reveals long chromosomal regions fixed for one allele. Our findings have some similarities with a study of the cattle genome [5] which consists mainly of a mosaic of haplotype blocks, i.e. regions with low haplotype diversity and high levels of linkage disequilibrium [5]. These haplotype blocks may reflect several factors, including genetic hitchhiking, variable mutation rates and recombination, gene flow, drift, inbreeding and lack of genetic equilibrium [6]. Therefore, the processes underlying these haplotype blocks should be interpreted with caution. However, it is likely that many of the shared blocks and, in turn, highly polymorphic regions are ancestral. Hence, more detailed screening could answer important evolutionary questions.

Very few segregating SNPs were shared between EB and PB (1.44%) and EB and WB (1.89%) when compared to the overlap between PB and WB (41.91%). The number of shared polymorphisms is inversely related to the estimated F_ST _values, confirming that they are related to the time elapsed since divergence in isolation of EB, PB and WB.

Simulations made with the software BOTTLENECK 1.2.02 [7] assuming an infinite alleles mutation model, showed decreased abundance of low frequency alleles (< 0.1) relative to intermediate allele frequency classes (0.1-0.2, 0.2-0.3, 0.3-0.4) in WB and PB, whereas for EB the distribution was L-shaped. This mode-shift in the distribution of allele frequencies in WB and PB signifies population bottlenecks. The tests indicate therefore that WB and PB are not in genetic equilibrium (as expected after recent demographic declines). However, the EB population is in genetic equilibrium, despite the documented recent, strong bottleneck. This unexpected result could be due to the fact that the bottleneck in EB may have reduced N_e_, which is negatively correlated to the speed at which the population re-establishes mutation-drift equilibrium and show therefore an L-shaped distribution. Given the distortion of the L-shaped distribution is transient, and likely to be detectable until genetic drift begin to re-establish mutation-drift equilibrium [7] we hypothesize that the extremely small N_e _produced by the drastic bottleneck in EB, has allowed a rapid return of the population to genetic equilibrium.

As a consequence of the finding that WB and PB are not at genetic equilibrium we must expect that the relation between F_ST _and divergence time between EB, WB and PB can be strongly biased. Additionally, the presence of blocks in the genome can probably partially be explained by this lacks of genetic equilibrium. Even if the EB population is now at equilibrium, we cannot exclude that previous bottlenecks have contributed to the observed pattern of a mosaic of haplotype blocks.

The presence of a mosaic blocks pattern and evidences for a mode-shift in the distribution of allele frequencies found in WB and PB agrees with theoretical expectations for populations which have undergone a severe bottleneck [8]. The finding that P% in EB is relatively smaller than P% PB and WB, is not surprising as the European bison underwent the most extreme bottleneck in population size of the three groups at the beginning of the last century; EB stems from only 7 animals [8]. Consequently, due to the founder effect, the current free-living Białowieża population of the European bison (about 400 individuals in 2007) is expected to have lower genetic diversity than the historic population. PB and WB have also experienced bottlenecks [9,10], of about 150 individuals in PB [11] and 250 in WB [12]. During the last 100 years the population size has increased and census population sizes are now estimated to be large both for PB and WB, although most of the bison in North America occur in commercial herds [10]. Despite rapid population growth in the last century, N_e _has therefore increased slowly in bison. This is because the long term N_e _is a function of the harmonic mean which is strongly influenced by the minimum population size reached [13,14].

In order to detect regions of bison chromosomes which show signs of positive selection we checked for the existence of "outlier SNPs" by creating distributions of H_E _for every locus in EB, PB and WB. The SNPs were considered outliers if their H_E _was > mean H_E _+ 3 SD or < mean H_E _- 3SD. These tests were performed pooling all loci and testing every single chromosome singularly. Additionally, we tested (with a *t*-test) for EB, WB and PB if the H_E _estimated from the polymorphic SNPs shared among EB, WB and PB were significantly different from the H_E _estimated from the unshared polymorphic SNPs. These tests were conducted because we suspected that the reasons for this shared polymorphism could be due to stabilizing selection. However, no significant differences were found. The lack of evidence for positive selection in EB, WB and PB is not surprising as the selection intensity has to be larger than  for selection to be efficient [15,16]. Given that N_e _is low for bison in general, all SNPs, even if not selectively neutral, behave neutrally until N_e _reaches a certain threshold.

By screening a large number of SNPs we considerably reduce the sampling error of the estimated overall genomic variability compared to traditional investigations that use a much more limited number of markers (SNPs or microsatellites). The SNPs can also be used in more limited numbers for genetic monitoring of populations. For many years, microsatellites have been the markers of choice for this purpose, using non-invasive techniques like extraction of DNA from scat or other organic material, and also using historical samples in order to add a temporal dimension to the survey. However, non-invasive DNA samples are often of poor quality and prone to genotyping errors due to DNA degradation, making long genetic markers like microsatellites difficult to use. Conversely, SNPs have the advantage that they are able to amplify short DNA sequences, decreasing therefore artifacts occurring due to degraded DNA. Additionally, the information from the BeadChip might allow genome-wide based breeding schemes designed to preserve rare alleles and minimize inbreeding (by estimating the true relationships between individuals). Traditional methods for making breeding decisions to reduce the level of inbreeding (by increasing N_e_) utilize only pedigree information, which describes the expected relationship among individuals. With the same pedigree, however, individuals still vary in the realized genetic relationship among them [17]. Therefore, information obtained from genetic markers can be useful in this respect as they will provide the realized genetic relationships.

The fact that the genome of EB, WB and PB is organised in blocks allows for designing breeding strategies which can use genotype data instead of pedigree data and which have the scope to increase or reduce the polymorphism of the blocks. The fact that there are several polymorphic SNPs embedded in the blocks (see fig. 1, 2 and 3), allow us to reduce considerably the number of SNPs which need to be screened in order to apply a breeding strategy. In fact, our results suggest that one single SNP can detect the level of polymorphism of an entire block. Hence, choosing a subset of the polymorphic SNPs (48 or 96 SNPs) we can apply relatively cheap technologies such as VeraCode or Fluidigm EP1 system (). With the information obtained from the BeadChip it will be possible to create a SNP panel on the polymorphisms described here and use them in a MAS strategy [18].

Usage of a SNP panel as proposed here would allow not only reliable parentage and identity analysis but would also provide a standardized panel for exchange of genotype data between laboratories. As the range of off-the-shelf SNP genotyping systems grows, they may find increased use for a number of endangered species (and rare domestic breeds). Further research is needed to determine if existing SNP genotyping systems can be applied across whole taxonomic orders or families, or if they are only transferable at the species or genus level, and to what extent screening success depends on taxonomic distance from the source taxon or population history of the study taxon. A problem often associated with SNPs application in population studies is ascertainment bias [19,20]. It is generated by heterogeneity in the SNP discovery process, variable sample size and composition, and may cause underestimation or overestimation of globally distributed SNP frequencies [20]. Since the chip markers used in our survey were selected for cattle, the possible ascertainment bias could appear when comparing the SNP based genetic variability of cattle and bison. Thus, a bison - cattle comparisons should be interpreted with caution. However the random selection of markers should not constitute a problem when the European bison is the only subject of an inquiry thus it was not considered in our study.

## List of abbreviations

EB: European bison; H_E_: expected heterozygosity; MAF: Minor allele frequency; MAS: Marker assisted selection; N_e _: Effective population size; P%: Percent of polymorphic loci; PB: Plain bison; S: Selection gradient; WB: Wood bison

## Competing interests

The authors declare that they have no competing interests.

## Authors' contributions

All authors have conceived the study and drafted the manuscript. All authors have read and approved the final manuscript.

## Reviewers' comments

### Reviewers' report 1

Fyodor Kondrashov (Centre for Genomic Regulation Barcelona Spain).

Reviewers' comments

This reviewer provided no comments for publication

### Reviewers' report 2

Shamil Sunyaev (Harvard Medical School, Boston MA, United States).

Reviewers' comments

This is an interesting study of genetic variation in bison. The authors characterized variation in the European bison and American wood and plains bisons using the BovineSNP50 Illumina Genotyping BeadChip. I have three comments listed below: 1)The manuscript seems to suggest that the observed small number of SNPs from the BovineSNP50 chip segregating in the bison population reflects a population bottleneck. However, the authors measured the level of shared polymorphism between bison and cow rather than the overall levels of variation in bison. The level of shared polymorphism has a more complex relationship with N_e _and also depends on other parameters (for the theoretical treatment see Clark AG. Neutral behaviour of shared polymorphism. Proc Natl Acad Sci U S A. 1997 94:7730-4.) Thus, a more sophisticated discussion of the observations would be appropriate instead of simply stating the low level of variation and, subsequently, N_e_.

*Author's response*

*We agree with the Reviewer that it would have been interesting to estimate the N_e _following the method presented in Clark (1997). This method is based on the fact that the loss of a shared polymorphism of a neutral allele having an initial allele frequency of 1/2 occurs sooner than loss of polymorphism in a single species and has an expected time of 1.7 N_e _generations, compared to 2.77N_e_, which is the mean time necessary for one neutral allele to undergo fixation within a single population. Therefore, given the fact that in our study we have found several shared polymorphisms, we would have been able to estimate the historical N_e _of EB, WB and PB. However, the method presented by Clark requires that the loci utilised for the estimation of N_e _are unlinked. Hence, we performed tests for linkage disequilibrium and ran the program Haploview 4.1 which showed that the genome of the bison is mainly a mosaic of haplotype blocks where strong linkage disequilibrium between SNPs is observed (see please the last paragraph on page 5 and please see fig. *1, 2* and *3* where the blocks are shown graphically). Therefore, we have not been able to use Clark's method, but we decided to utilise the shared polymorphism in order to test if H_E _within a subspecies (pooling all the SNPs with shared polymorphism) was significantly different from H_E _within a subspecies (pooling all the SNPs which are not shared among EB, WB and PB). The differences of mean H_E _was not significant. Therefore, we did not obtain evidence for the fact that the shared polymorphism could be due to stabilizing selection acting on these SNPs (please see the first paragraph on page 8)*.

2) The manuscript would benefit from the analysis of allele frequency distribution (excess of high frequency variants may be suggestive of a bottleneck) and F_ST _between the analyzed populations.

Author's response

*Population bottlenecks cause a characteristic mode-shift distortion in the distribution of allele frequencies at selectively neutral loci. In a stable population the distribution is expected to be L-shaped. Bottlenecks cause alleles at low frequency (< 0.1) to become less abundant than alleles in more intermediate allele frequency classes (e.g., 0.1-0.2). We performed simulations with the software BOTTLENECK 1.2.02, assuming an infinite allele mutation model searching for mode-shift distortion in the distribution of allele frequencies. We found evidence for lack of genetic equilibrium in WB and PB (see please the third paragraph on page 5). Furthermore, the pairwise F_ST _values as a measure of differentiation between EB, PB and WB have been added (see please the first paragraph on page 5)*.

3) Is it possible to quantify the improvement of breeding strategies using genotyping instead of pedigree data?

*Author's response*

*An exact quantification of the improvement of breeding strategy can only be made using empirical data comparing the improvement of a certain trait in mating individuals chosen on the basis of their pedigree or on the basis of their genetic similarity at the molecular level. Traditional methods for making breeding decisions to reduce the level of inbreeding (by increasing N_e_) utilize only pedigree information, which describes the expected relationship among individuals. With the same pedigree, however, individuals still vary in the realized genetic relationship between them. Therefore, information obtained from genetic markers can be useful in this respect as they will provide the realized genetic relationships. Additionally, information from genome-wide screening also enables detection of genes associated with hereditary genetic diseases and inbreeding depression (these issues have been discusses in the last paragraph on page 8)*.
